# Supplementary material for: Facile Synthesis to Porous TiO2 Nanostructures at Low Temperature for Efficient Visible-Light Degradation of Tetracycline
Source: Nanomaterials (Basel). 2024 May 27;14(11):943. doi: 10.3390/nano14110943 (PMC11173820; doi:10.3390/nano14110943)
Supplement: Supplementary file 1 [file nanomaterials-14-00943-s001.zip › nanomaterials-2996493-supplementary.pdf]

Supplementary Material

Facile Synthesis to Porous TiO_2 Nanostructures at Low Temperature for Efficient Visible-light Degradation of Tetracycline

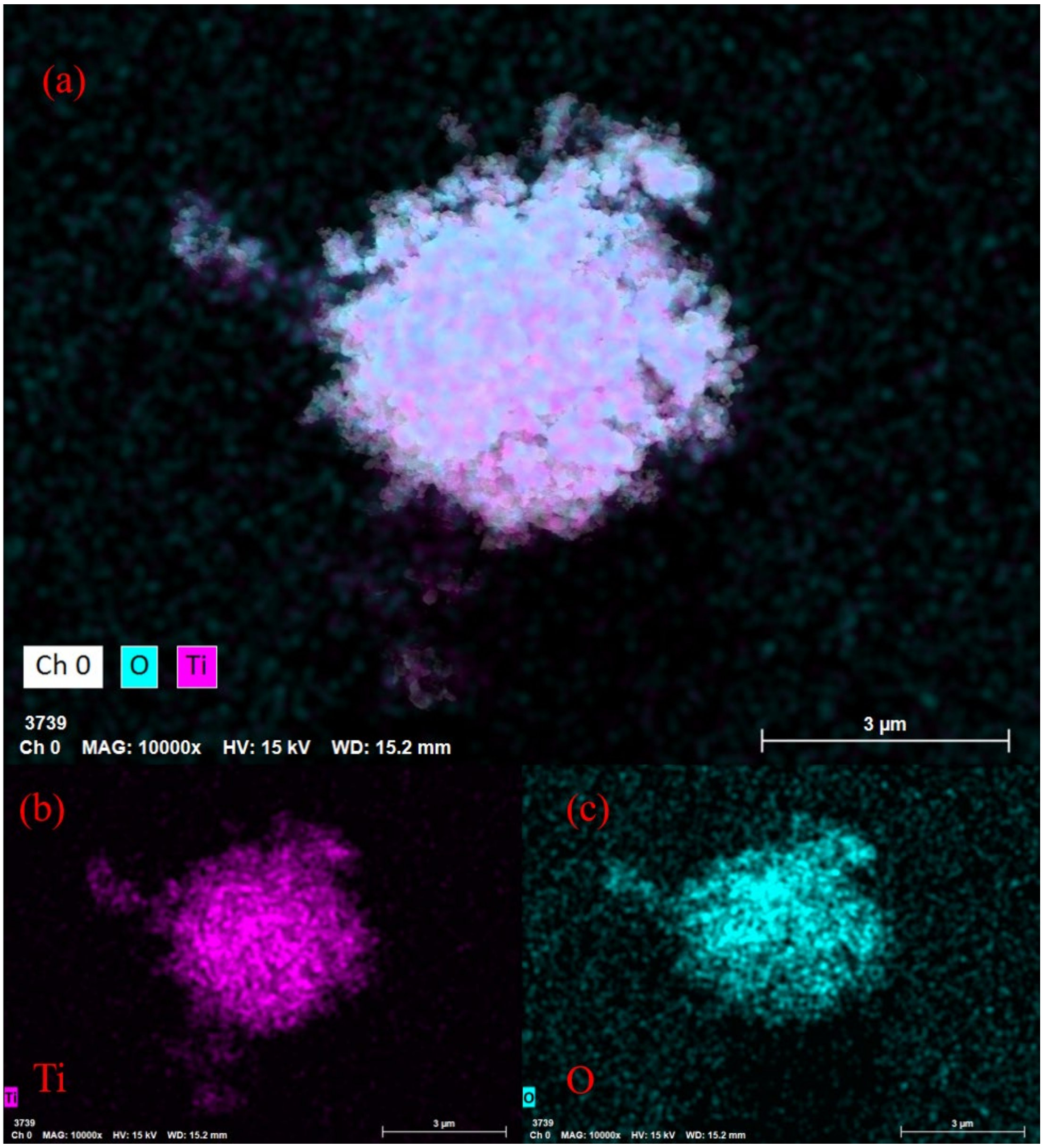

Fig. S1 Elements mapping of TiO_2-100

Table S1 Specific surface areas, pore volume, and pore diameter of these samples.

Samples	Surface Area(m^2/g)	BJH Pore Volume(cm^3/g)	BJH Average Pore Size(nm)
TiO_2-90	33	0.13	7.96
TiO_2-100	57	0.18	9.07
TiO_2-110	40	0.16	9.32
P25	9.2	0.031	5.98

Table S2 The pseudo-first-order kinetic rate constants of different samples

Samples	UV Light		Visible Light	
	Kinetic rate constants (k, min^{-1})	Correlation coefficients (R^2)	Kinetic rate constants (k, min^{-1})	Correlation coefficients (R^2)
TiO_2-90	0.09047	0.95691	0.01684	0.98666
TiO_2-100	0.18515	0.98452	0.02167	0.97614
TiO_2-110	0.10482	0.98241	0.01836	0.95829
P25	0.00967	0.97868	0.00505	0.98623

Table S3 Comparison of removal efficiency of various photocatalysts toward TC

Photocatalyst	light type	Light time (min)	Degradation rate (%)	References
TiO_2-GC	visible light	120	94.6	[1]
TiO_2/Ag	visible light	90	90	[2]
P25	UV/visible	120	94.8/76.6	[3]
black anatase-TiO_2	light	270	66.2	[4]
TiO_2@MoS_2/$BiVO_4$	visible light	90	72.3%	[5]
hollow TiO_2	visible light	120	90	[6]

PG/TiO_2	UV/visible light	90	95.5/88.6	[7]
TiO_2/RM	visible light	120	60	[8]
$BiVO_4/TiO_2/RGO$	visible light	120	96.2	[9]
TiO_2	UV/ visible light	60	Nearly100/79	This work

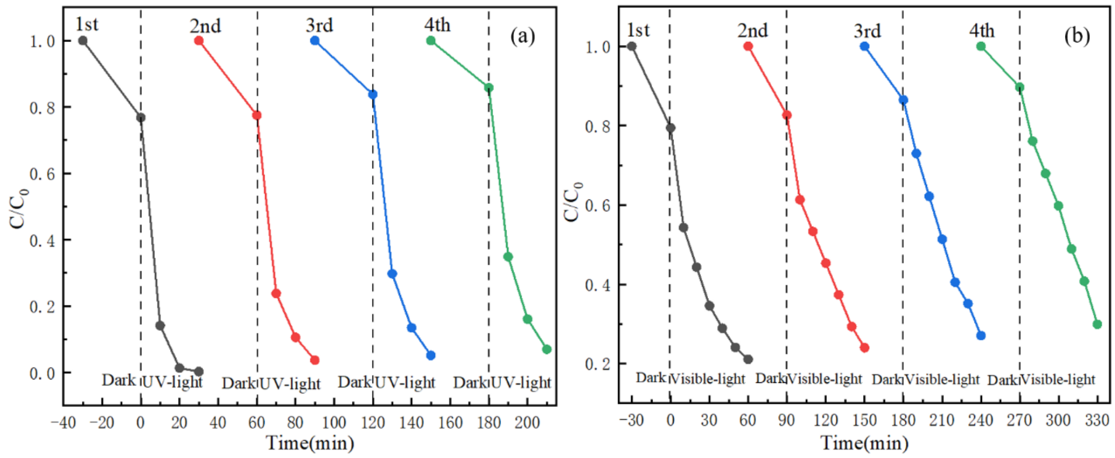

Fig. S2 Recycled testing of TiO2-100 for TC(a) UV-light;(b) visible light.

Table S4 Fukui Function indices of atoms in TC

Number	Atom	q_N	q_{N+1}	q_{N-1}	f^-	f^+	f^0
1	O	-0.2227	-0.2414	-0.194	0.0288	0.0187	0.0237
2	C	-0.1005	-0.107	-0.0961	0.0045	0.0065	0.0055
3	O	-0.2201	-0.2525	-0.1967	0.0235	0.0324	0.0279
4	O	-0.1658	-0.2179	-0.1026	0.0633	0.0521	0.0577
5	O	-0.1584	-0.1816	-0.1229	0.0356	0.0232	0.0294
6	O	-0.2228	-0.3048	-0.191	0.0319	0.082	0.0569
7	O	-0.32	-0.3385	-0.2111	0.1089	0.0186	0.0637
8	N	-0.1577	-0.1677	-0.1225	0.0352	0.01	0.0226
9	O	-0.1398	-0.1667	-0.1201	0.0197	0.0269	0.0233
10	O	-0.2085	-0.222	-0.1974	0.0111	0.0135	0.0123
11	C	-0.0363	-0.0405	-0.0321	0.0042	0.0041	0.0042
12	C	-0.0344	-0.0378	-0.0305	0.0039	0.0034	0.0037
13	C	0.136	0.1201	0.1395	0.0035	0.0159	0.0097
14	C	0.1522	0.149	0.1799	0.0277	0.0032	0.0154
15	C	0.1055	0.0509	0.1433	0.0378	0.0546	0.0462
16	C	0.0962	0.0647	0.1226	0.0263	0.0316	0.029
17	C	0.1286	0.05	0.1419	0.0133	0.0785	0.0459

18	C	0.0647	0.0583	0.0679	0.0033	0.0064	0.0048
19	C	-0.0735	-0.0815	-0.0628	0.0107	0.0081	0.0094
20	C	-0.0742	-0.0879	0.0055	0.0798	0.0137	0.0467
21	C	-0.0378	-0.0586	-0.0201	0.0178	0.0208	0.0193
22	C	-0.0732	-0.1101	-0.0493	0.0239	0.0369	0.0304
23	C	0.1254	0.1037	0.136	0.0106	0.0217	0.0161
24	C	-0.0283	-0.0305	-0.027	0.0013	0.0022	0.0017
25	C	-0.0228	-0.0255	-0.0132	0.0096	0.0027	0.0062
26	C	0.0052	-0.0296	0.0087	0.0035	0.0348	0.0191
27	C	-0.0269	-0.0961	0.0078	0.0347	0.0692	0.052
28	C	-0.0645	-0.092	-0.0325	0.0319	0.0275	0.0297
29	C	0.0205	0.0192	0.0233	0.0028	0.0013	0.002
30	C	-0.05	-0.0547	-0.0445	0.0055	0.0047	0.0051
31	C	0.0857	0.0831	0.0884	0.0027	0.0026	0.0026
32	N	-0.049	-0.0505	-0.0455	0.0035	0.0015	0.0025

Table S5 Possible major intermediates in the TC degradation process

Product ID	Chemical Formula	Proposed Structure	Measured (m/z)
TC	$C_{22}H_{24}N_2O_8$	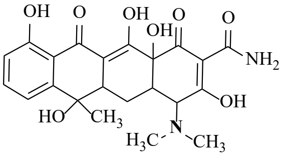	445
P1	$C_{20}H_{20}N_2O_8$	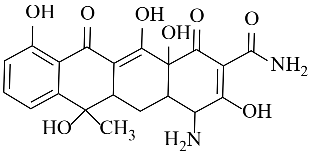	417
P2	$C_{22}H_{25}N_2O_9$	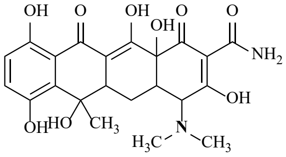	462
P3	$C_{20}H_{21}N_2O_9$	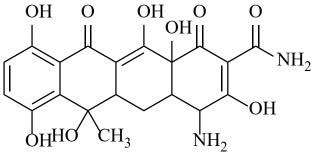	434
P4	$C_{20}H_{17}O_8$	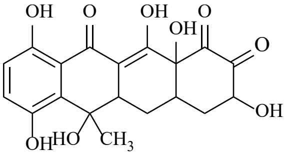	402

P5	$C_{20}H_{17}O_8$	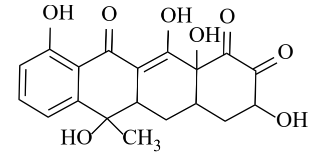	385
P6	$C_{20}H_{11}O_3$	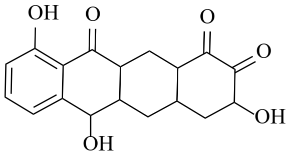	340
P7	$C_{19}H_8O_3$	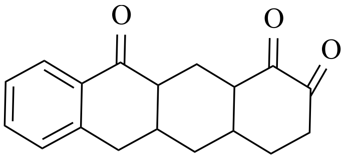	285
P8	$C_{12}H_{20}O_7$	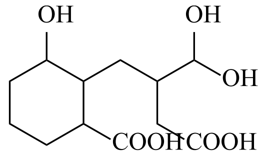	277
P9	$C_2H_4O_2$	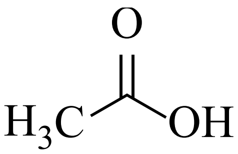	60
P10	$C_3H_2O_5$	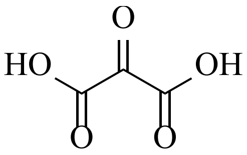	118

References

1 Dong, D.; Wang, K.; Yi, M.; Liang, Y.; Muhammad, Y.; Wei, E.; Wei, Y.; Fujita T. Preparation of TiO_2 photocatalyst microspheres by geopolymer technology for the degradation of tetracycline. J. Clean. Prod., **2022**,339,130734.

2 Gang, R.; Xia, Y.; Xu, L.; Zhang, L.; Ju, S.; Wang, Z.; Koppala, S. Size controlled Ag decorated TiO_2 plasmonic photocatalysts for tetracycline degradation under visible light. Surf. Interfaces, **2022**,31,102018.

3 Wu,S.; Hu, H.; Lin, Y.; Zhang, J.; Hu, Y.H. Visible light photocatalytic degradation of tetracycline over TiO_2. Chem. Eng. J. **2020**,382, 122842.

4 Wu,S.; Li, X.; Tian, Y.; Lin, Y.; Hu, Y. H. Excellent photocatalytic degradation of tetracycline over black anatase-TiO_2 under visible light. Chem. Eng. J., **2021**,406,126747.

5 Raj, M. G.; Vijayakumar, E.; Preetha, R.; Narendran, M. G.; Neppolian, B.; Bosco, A. J. Implanting TiO_2 @MoS_2/$BiVO_4$ nanocomposites via sonochemically assisted photoinduced charge carriers promotes highly efficient photocatalytic removal of tetracycline. J. Alloys Compd., **2022**,929,167252.

6 Zhang, S.; Yin, Z.; Xie, L.; Yi, J.; Tang, W.; Tang, T.; Chen, J.; Cao, S. Facet engineered TiO_2 hollow sphere for the visible-light-mediated degradation of antibiotics via ligand-to-metal charge transfer. Ceram. Int., **2020**,46, 8949-8957.

7 Zhang, N.; Ning, X.; Chen, J.; Xue, J.; Lu, G.; Qiu, H. Photocatalytic degradation of tetracycline based on the highly reactive interface between graphene nanopore and TiO_2 nanoparticles. Micropor. Mesopor. Mat., **2022**,338,111958.

8 Meng, F.; Ling, Y.; Li, Y.; Liu, D.; Wei, K.; Sun, L.;Sang, Z. Synthesis of visible-light-driven photocatalyst of TiO_2 modified waste red mud and its application in tetracycline hydrochloride removal. Surf. Interfaces, **2022**,35,102482.

9 Wang, W.; Han, Q.; Zhu, Z.; Zhang, L.; Zhong, S.; Liu, B. Enhanced photocatalytic degradation performance of organic contaminants by heterojunction photocatalyst $BiVO_4$/TiO_2/RGO and its compatibility on four different tetracycline antibiotics. Adv. Powder Technol., **2019**,30, 1882-1896.
